# Disinfectant-susceptibility of multi-drug-resistant *Mycobacterium tuberculosis* isolated in Japan

**DOI:** 10.1186/s13756-016-0102-y

**Published:** 2016-02-08

**Authors:** Noriko Shinoda, Satoshi Mitarai, Eri Suzuki, Mineo Watanabe

**Affiliations:** Graduate School of Infection Control Sciences, Kitasato University, 5-9-1 Shirokane, Minato-ku, Tokyo 108-8641 Japan; Research Institute of Tuberculosis, Japan Anti-Tuberculosis Association, 3-1-24 Matsuyama, Kiyose, Tokyo 204-8533 Japan; Laboratory of Medical Microbiology, Kitasato Institute for Life Sciences, Kitasato University, 5-9-1 Shirokane, Minato-ku, Tokyo 108-8641 Japan

**Keywords:** Multi-drug-resistant *Mycobacterium tuberculosis*, Disinfectant, Microbicide

## Abstract

**Background:**

Multi-drug-resistant *Mycobacterium tuberculosis* has been an important problem in public health around the world. However, limited information about disinfectant-susceptibility of multi-drug-resistant strain of *M. tuberculosis* was available.

**Findings:**

We studied susceptibility of several Japanese isolates of multi-drug-resistant *M. tuberculosis* against disinfectants, which are commonly used in clinical and research laboratories. We selected a laboratory reference strain (H37Rv) and eight Japanese isolates, containing five drug-susceptible strains and three multi-drug-resistant strains, and determined profiles of susceptibility against eight disinfectants. The *M. tuberculosis* strains were distinguished into two groups by the susceptibility profile. There was no relationship between multi-drug-resistance and disinfectant-susceptibility in the *M. tuberculosis* strains. Cresol soap and oxydol were effective against all strains we tested, regardless of drug resistance.

**Conclusions:**

Disinfectant-resistance is independent from multi-drug-resistance in *M. tuberculosis*. Cresol soap and oxydol were effective against all strains we tested, regardless of drug resistance.

## Findings

Tuberculosis is still a major cause of death in low to middle-income countries and areas [1]. Furthermore, drug resistant and multi-drug-resistant tuberculosis has been reported worldwide [2]. Tuberculosis has remained as a major public health threat even in this century [1, 2].

Disinfectants are widely used to eliminate infectious agents from possibly contaminated equipment and specimens. Effectiveness of disinfectants against *M. tuberculosis* was reported previously [3–5]. However, poor information about disinfectant effectiveness against multi-drug-resistant (MDR) strain of *M. tuberculosis* is available. In this study, we first evaluated effectiveness of disinfectants against MDR-*M. tuberculosis* in just short time (1-min), on the supposition that the disinfectants were used for routine environmental cleaning by spray, wipe, or wash in relatively short time to *M. tuberculosis*. Then we discussed relationship between drug resistance and disinfectant resistance in *M. tuberculosis*.

*M. tuberculosis* strains used in this study are listed in Table 1. *M. tuberculosis* strain H37Rv is the reference strain isolated in US in 1934. Other eight strains were fresh clinical isolates from Japanese patients. The strains were cultured in Middlebrook 7H9 broth (BD Biosciences, Sparks, MD) supplemented with 10 % OADC Enrichment (BD Biosciences) and 0.05 % (w/v) Tween 80 (this medium was referred as MB broth).

**Table 1:** *M. tuberculosis* strains used in this study

Strain	Place	Year	Multidrug resistance	Disinfectant							
				ADEG	CG	PI	BK	OX	CS	EtOH	BK + EtOH
H37Rv	US	1934	Type strain	+	+	+	+	+	+	+	+
2A-3-6	Japan	2002	–	–	–	–	–	+	+	–	–
2E-1-9	Japan	2002	–	+	+	+	+	+	+	–	–
2U-5-12	Japan	2002	–	–	–	+	–	+	+	–	–
2U-11-2	Japan	2002	–	+	+	+	+	+	+	+	–
2Z-1-3	Japan	2002	–	+	–	–	–	+	+	–	–
LV-15	Japan	2008	INH, RFP, SM, EB, LVFX	–	+	+	+	+	+	–	–
LV-36	Japan	2010	INH, RFP, SM	–	–	–	–	+	+	–	+
LV-79	Japan	2009	INH, RFP, SM, LVFX	+	+	+	+	+	+	+	+

*INH*, isoniazid; *RFP*, rifampicin; *SM*, streptomycin; *EB*, ethambutol; and *LVFX*, levofloxacin, *ADEG* alkyldiaminoethylglycine-HCl (0.2 % W/V); *CG* chlorhexidine gluconate (0.1 % W/V); *PI*, povidine iodine (10 mg/ml as active iodine); *BK* benzalkonium-HCl (0.1 % W/V); *OX* oxydol (3 % W/V); *CS* cresol soap (2 % V/V); *EtOH* ethanol (70 % V/V); and *BK + EtOH* benzalkonium-HCl (0.1 % W/V) + ethanol (70 % V/V)

+: the disinfectant inhibited growth of the bacteria (effective disinfectants)

−: the disinfectant did not inhibit growth of the bacteria (not effective)

We selected commonly used and easily available eight disinfectants, including 0.2 % (W/V) alkyldiaminoethylglycine-HCl (ADEG), 0.1 % (W/V) chlorhexidine gluconate (CG), 10 mg/ml povidine iodine (PI), 0.1 % (W/V) benzalkonium-HCl (BK), 3 % (W/V) oxydol (OX), 2 % (V/V) cresol soap (CS), 70 % (V/V) ethanol (EtOH), and 0.1 % (W/V) benzalkonium-HCl + 70 % (V/V) ethanol (BK + EtOH). The disinfectants were diluted with distilled water according to the instructions of the manufacturers. *M. tuberculosis* strains were cultured in MB broth for 14 days at 37 °C. Optical density at 650 nm (OD_650_) of each bacterial culture was adjusted to 1.0. The adjusted bacterial culture was diluted to 0.1 of OD_650_ with fresh MB broth, and then its 100 μl was added into 1 ml of diluted disinfectant in a sterilized tube with screw cap. After 30 s incubation at room temperature, the tube was centrifuged for 30 s at 9600 × g at room temperature and the supernatants were removed. The pellet was immediately resuspended in 1 ml of fresh MB broth, and then the suspension was cultured for 14 days at 37 °C. The 14 days culture was sufficient to detect growth of *M. tuberculosis* by the real time qPCR described below. The tubes were centrifuged (16,200 × g, 2 min, room temperature) to collect bacterial cells. Then the cells were suspended in solution containing 20 μl of 0.5 M NaOH, 4 μl of 10 % sodium dodecyl sulfate, and 180 μl of distilled water. The suspension was heated at 95 °C for 15 min, and then cooled to room temperature. Two hundred microliters of phenol/chloroform (1:1) was added and then mixed strongly. After centrifugation at 16,200 × g for 5 min, its aqueous phase was transferred into a new tube, and this step was repeated twice. The aqueous phase was added with 16 μl of 5 M NaCl and 800 μl of 70 % ethanol, and then centrifuged at 16,200 × g for 1 min. The pellet (purified total DNA) was resuspended in 50 μl of distilled water.

Bacterial growth was measured 16S rRNA gene-targeted real-time quantitative PCR (qPCR) with the 16S rRNA-TF primer (5′-ACGGAAAGGTCTCTTCG-3′) and 16S rRNA-TR primer (5′- GTCGTCGCCTTGGTAG-3′) [6]. The PCR was performed by using KAPA SYBR FAST qPCR Master Mix (2×) Universal (NIPPON Genetics, Tokyo, Japan) according to the manufacture’s instruction. Real-time qPCR was performed using the following cycling conditions: 1 cycle of 95 °C for 30 s, 40 cycles of 95 °C for 5 s and 60 °C for 30 s, a final extension of 60 °C for 5 s and a melting curve of 60 °C to 95 °C.

Efficacy of disinfectant was evaluated by growth inhibition of the bacteria. Disinfectants were judged as not effective when the copy number of 16S rRNA gene would be significantly increased by culture. One-way ANOVA with Dunnett’s test was used for the statistical analysis.

*M. tuberculosis* strain H37Rv, which is a widely used laboratory strain, was susceptible to all the disinfectants we tested. Since the strain was isolated in 1934 [7], it could be adapted to laboratory propagations. The strain might lose resistance against bactericidal compounds.

**Fig. 1:**
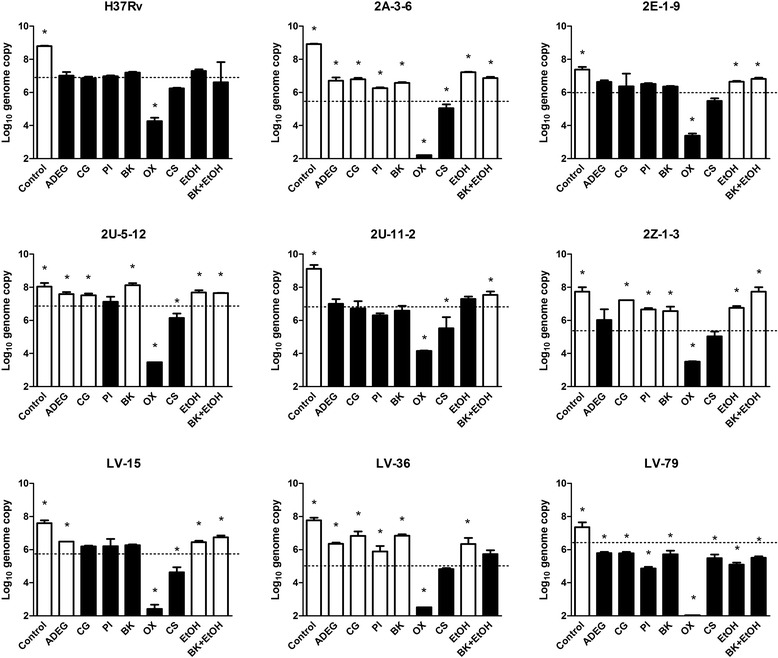
Efficacy of disinfectants to *M. tuberculosis* stains. Each *M. tuberculosis* stain was exposed to the disinfectants for 1 min at room temperature. The bacteria were incubated in Middlebrook 7H9 broth for 14 days at 37 °C. Bacterial growth was measured by 16S rRNA targeted real-time qPCR. Control was not exposure by any disinfectants. Dashed line indicates the genome copy number on the day of inoculation. ADEG, alkyldiaminoethylglycine-HCl (0.2 % W/V); CG, chlorhexidine gluconate (0.1 % W/V); PI, povidine iodine (10 mg/ml as active iodine); BK, benzalkonium-HCl (0.1 % W/V); OX, oxydol (3 % W/V); CS, cresol soap (2 % V/V); EtOH, ethanol (70 % V/V); and BK + EtOH, benzalkonium-HCl (0.1 % W/V) + ethanol (70 % V/V). The results are expressed as means ± SD. *: *p* <0.05 (v.s. genome number on the day of inoculation). Black bars: the disinfectant inhibited growth of *M. tuberculosis* (effective disinfectant). White bars: the disinfectant did not inhibit growth of the bacteria (not effective)

We found that there were two groups in the Japanese isolates of *M. tuberculosis* based on susceptibility against disinfectants. Strain 2E-1-9, 2U-11-2, LV-15, and LV-79 were susceptible to most disinfectants used in this study, whereas the strain 2A-3-6, 2U-5-12, 2Z-1-3, and LV-36 showed resistance against most disinfectants except oxydol and cresol soap (Fig. 1, Table 1). There was no relationship between multi-drug-resistance and disinfectant-resistance in the selected *M. tuberculosis* strains. The tick and waxy cell wall of *M. tuberculosis* is assumed to act as a major barrier to penetration of antibiotics and disinfectants [8], and could affect to resistance against both antibiotics and disinfectants. Since there was no correlation between drug resistance and disinfectant resistance to the *M. tuberculosis* strains in this study, the drug resistance and the disinfectant resistance might be based on different mechanism in the strains.

Oxydol and cresol soap were effective against all *M. tuberculosis* strains tested in this study, even MDR strains. Our results suggested that more than 1 min treatments with oxydol or cresol soap were promising to eliminate contamination of *M. tuberculosis*, regardless of drug resistance. However, it is known that disinfectants could decrease their efficacy by organic compounds, for example, blood, sputum, and other dirt. It should be noticed that disinfectant could be used carefully against *M. tuberculosis* in clinical specimens and things that contain organic compounds.
